# Kinase-targeted cancer therapies: progress, challenges and future directions

**DOI:** 10.1186/s12943-018-0804-2

**Published:** 2018-02-19

**Authors:** Khushwant S. Bhullar, Naiara Orrego Lagarón, Eileen M. McGowan, Indu Parmar, Amitabh Jha, Basil P. Hubbard, H. P. Vasantha Rupasinghe

**Affiliations:** 1 Department of Pharmacology, Faculty of Medicine and Dentistry, University of Alberta, Edmonton, AB Canada; 2 Department of Pharmacy and Pharmaceutical Technology, Faculty of Pharmacy, University of Barcelona, Barcelona, Spain; 3 Chronic Disease Solutions Team, School of Life Science, University of Technology, New South Wales, Australia; 4 Division of Product Development, Radient Technologies, Edmonton, AB Canada; 5 Department of Chemistry, Acadia University, Wolfville, NS Canada; 6 Department of Plant, Food, and Environmental Sciences, Faculty of Agriculture, Dalhousie University, Truro, NS Canada; 7 Department of Pathology, Faculty of Medicine, Dalhousie University, Halifax, NS Canada

**Keywords:** Kinases, Kinase inhibition, Small-molecule drugs, Cancer, Oncology

## Abstract

The human genome encodes 538 protein kinases that transfer a γ-phosphate group from ATP to serine, threonine, or tyrosine residues. Many of these kinases are associated with human cancer initiation and progression. The recent development of small-molecule kinase inhibitors for the treatment of diverse types of cancer has proven successful in clinical therapy. Significantly, protein kinases are the second most targeted group of drug targets, after the G-protein-coupled receptors. Since the development of the first protein kinase inhibitor, in the early 1980s, 37 kinase inhibitors have received FDA approval for treatment of malignancies such as breast and lung cancer. Furthermore, about 150 kinase-targeted drugs are in clinical phase trials, and many kinase-specific inhibitors are in the preclinical stage of drug development. Nevertheless, many factors confound the clinical efficacy of these molecules. Specific tumor genetics, tumor microenvironment, drug resistance, and pharmacogenomics determine how useful a compound will be in the treatment of a given cancer. This review provides an overview of kinase-targeted drug discovery and development in relation to oncology and highlights the challenges and future potential for kinase-targeted cancer therapies.

## Background

Kinases are enzymes that transfer a phosphate group to a protein while phosphatases remove a phosphate group from protein. Together, these two enzymatic processes modulate numerous activities of proteins in a cell, often in response to an external stimulus [1]. Approximately 538 known kinases are encoded in the human genome, and these kinases maintain cellular function by turning protein function on, while corresponding phosphatases reverse this action [2, 3]. These counter mechanisms greatly improve the plasticity of epigenome by regulating protein activity in virtually every imaginable way. Biochemically, protein kinases catalyze the following reaction [3]:

$$ {\mathsf{MgATP}}^{\mathsf{1}-}+\mathsf{protein}-\mathsf{O}:\mathsf{H}\to \kern0.75em \mathsf{protein}-\mathsf{O}:{{\mathsf{PO}}_{\mathsf{3}}}^{\mathsf{2}-}+\mathsf{MgADP}+{\mathsf{H}}^{+} $$

**Fig. 1:**
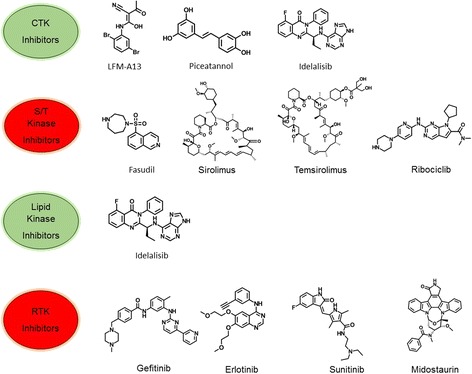
Chemical structures of representative kinase inhibitors used for treatment of various human cancers

Recent advances in our understanding of the fundamental molecular mechanisms underlying cancer cell signaling have elucidated a crucial role for kinases in the carcinogenesis and metastases of various types of cancer [4]. Since most protein kinases promote cell proliferation, survival and migration, when constitutively overexpressed, or active, they are also associated with oncogenesis [5]. Genome-wide studies of kinase mutations have revealed genetically inherited variants of specific kinases are causally associated with cancer initiation, promotion, progression as well as recurrence [4, 6]. Over the last three decades, multiple human malignancies have been identified to be associated with modulation and dysfunction of protein and lipid kinases and deactivated phosphatases on account of chromosomal reshuffling and genetic mutations [7–9]. Apart from the oncological issues, dysregulation of kinases has been demonstrated in many human disorders including immune, neurological and infectious diseases [10–13]. However, there is probably no greater clinical niche for kinases as the key targets for developing drugs than in cancer therapy. Kinome, the complete set of protein kinases encoded in its genome has become an attractive target for the treatment of numerous types of cancer. Single and multiple kinase inhibitors, both synthetic and natural molecules, are now targeted therapeutic strategies for treatment of human malignancies. The ROCK kinase inhibitor fasudil for treating cerebral vasospasms was the first approved small molecule for clinical use [14]. Kinase inhibitors now account for a quarter of all current drug discovery research and development efforts. Key oncogenic kinase drug targets include the PIK3CA, BRAF, and epidermal growth factor receptor (EGFR), which activates significant tumor cell signaling pathways and is related to the mutations and/or deletions in phosphatase and tensin homolog (PTEN), a phosphatase that negatively regulates PI3K [6, 7, 15]. Approximately 538 kinases are encoded in the human genome. Apart from this wide range of kinase-based drug targets, inhibition of distinct kinase signaling pathways can be less cytotoxic to non-cancerous cells, thus presenting the selective killing of tumor cells with considerably lower toxic manifestations [16, 17]. Interestingly, specific-kinase inhibitors, currently in clinical treatments, e.g., imatinib and dasatinib, produce more favorable outcome compared to conventional cytotoxic therapy [18, 19]. These kinase inhibitors have achieved a significant increase in patient survival rate in myeloid leukemia (CML) and gastrointestinal stromal tumors (GIST), thus translating basic molecular research into effective patient treatment. Due to improved clinical efficacy, U.S. Food and Drug Administration (FDA) has approved many small-molecule kinase inhibitors for clinical use (Fig. 1). These kinase inhibitors include target kinome members such as EGFR, ERBB2, VEGFRs, Kit, PDGFRs, ABL, SRC and mTOR, all providing improved clinical outcome and patient health status [4, 20]. The majority of these inhibitors target the ATP-binding site [21, 22], while a few of the non-ATP competitive kinase inhibitors target novel allosteric sites [23]. Consequently, the inhibition of kinase activity in treated patients prompts multiple anti-proliferative mechanisms, which leads to clinical remission of cancer.

The current procedure for developing robust and selective kinase inhibitors has swiftly evolved from synthesizing analogs of staurosporine to sophisticated structure-based design methodologies, facilitated by molecular docking, crystallography and nuclear magnetic resonance spectroscopy [24, 25]. Since 2001, more than 10,000 patent applications for kinase inhibitors have been filed in the United States alone. In addition to the small-molecule kinase inhibitors, kinase-targeted antibodies have also demonstrated efficacy in various cancers, for example, cetuximab in colorectal and head and neck cancer, and trastuzumab in breast cancer [26, 27]. Trastuzumab and cetuximab bind to the extracellular domain of HER2 and EGFR respectively, and block the binding of the natural ligand, thus avoiding conformational rearrangement essential to the activation of the kinase and its downstream kinase-signaling pathways. Currently, FDA has approved 35 drugs (31 for cancer therapy) including orally effective direct protein kinase inhibitors that target a limited number of enzymes (Table 1). However, despite these encouraging results, the problems with drug resistance, toxicity, and compromised efficacy present critical challenges in both clinical and experimental oncology [3]. Furthermore, problems in the synthesis of novel kinase inhibitors have plagued drug development through an inadequate understanding of the selectivity of the kinase inhibitors [16, 28]. A key challenge in the clinical assessment is to identify the most efficient combination of kinase targets and then develop treatment combinations for targeted cancer. These issues have prompted research initiatives that may override various limitation of kinase inhibition, particularly evading the treatment-related drug resistance. In this current review, the authors examined the status, novel methodologies of drug design and validation of the prospective kinase inhibitors for clinical usage.

**Table 1:** List of FDA-approved kinase inhibitors and their drug targets

Drug target	Protein substrate	Drug
ALK	Tyrosine	Crizotinib, Ceritinib, Alectinib, Brigatinib
BCR–Abl	Tyrosine	Bosutinib, Dasatinib, Imatinib, Nilotinib, Ponatinib
B-Raf	Serine/threonine	Vemurafenib, Dabrafenib
BTK	Tyrosine	Ibrutinib
CDK family	Serine/threonine	Palbociclib, Sorafenib, Ribociclib
c-Met	Tyrosine	Crizotinib, Cabozantinib
EGFR family	Tyrosine	Gefitinib, Erlotinib, Lapatinib, Vandetanib, Afatinib, Osimertinib
JAK family	Tyrosine	Ruxolitinib, Tofacitinib
MEK1/2	Dual specificity	Trametinib
PDGFR α/β	Tyrosine	Axitinib, Gefitinib, Imatinib, Lenvatinib, Nintedanib, Pazopanib, Regorafenib, Sorafenib, Sunitinib
RET	Tyrosine	Vandetanib
Src family	Tyrosine	Bosutinib, Dasatinib, Ponatinib, Vandetanib
VEGFR family	Tyrosine	Axitinib, Lenvatinib, Nintedanib, Regorafenib, Pazopanib, Sorafenib, Sunitinib

## Role of kinases in cancer

**Fig. 2:**
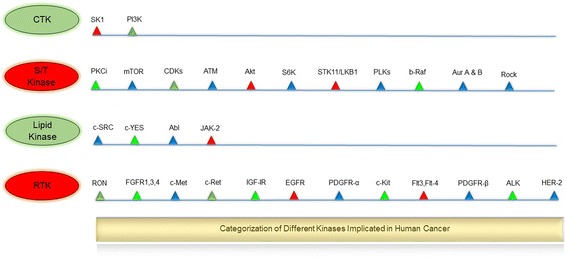
Categorization of different kinases implicated in human cancer. CTK: cytoplasmic tyrosine kinase, S/T Kinase: serine/threonine kinase, LK: lipid kinase, RTK: receptor tyrosine kinase. SK1: Sphingosine kinase 1, PI3K: phosphoinositide 3-kinase, PKCi: Protein kinase Ci, mTOR: mammalian target of rapamycin, CDKs: cyclin-dependent kinases, ATM: Ataxia telangiectasia mutated, Akt: protein kinase B, S6K: ribosomal protein S6 kinase, STK11/LKB1: Serine/threonine kinase 11 or liver kinase B1, PLKs: Polo-like kinases, b-Raf: B-Raf proto-oncogene, Aur A & B: Aurora Kinase A & B, c-SRC: Proto-oncogene tyrosine-protein kinase Src, c-YES: c-Yes proto-oncogene (pp62c-Yes), Abl: Abelson murine leukemia viral oncogene homolog 1, JAK-2: Janus kinase 2, RON: Recepteur d’Origine Nantais, FGFRs: Fibroblast growth factor receptors, c-Met: c-MET proto-oncogene, c-Ret: c-RET proto-oncogene, IGF-IR: Insulin-like growth factor 1 receptor, EGFR: Epidermal growth factor receptor, PDGFR-α: Platelet-derived growth factor receptor α, c-Kit: proto-oncogene c-Kit or Mast/stem cell growth factor receptor, Flt3,Flt-4: Fms-like tyrosine kinase 3, 4, PDGFR-β: Platelet-derived growth factor receptor β, ALK: Anaplastic lymphoma kinase, HER-2: human epidermal growth factor receptor-2

Targeting the kinases harboring oncogenic transformational capacity and metastasis has led to a notable change in the clinical management of cancer (Fig. 2). Hundreds of kinases play overlapping and intricate roles in cell transformation, tumor initiation, survival and proliferation. Diving kinases while justifying their coinciding functionalities is difficult. However, in order to understand and discuss their oncogenic undertakings, they can be vaguely categorized based on their hallmark roles in cancer. The first group is the kinases that play a fundamental role in the primary oncogenic transformation and thus present themselves as prospective drug targets. Cytoplasmic tyrosine kinases are critical conveyers of extracellular signals, and mutations in these kinases have been reported to occur in various oncogenic conditions. This category includes the PI3K family of dual specific protein/lipid kinases, which are the most frequently mutated kinases implicated in 30–50% of human cancers [29]. PI3KCA, perhaps the most notable member of PI3K family is associated with the pathology of colorectal cancer [30], breast cancer [31], ovarian cancer [32], endometrial carcinoma [33], and hepatocellular carcinoma [34]. The PI3KCA kinase catalyzes the production of PIP3, a phospholipid which activates downstream signaling components such as protein kinase AKT and promotes tumor cell growth and survival [35]. Similarly, active form of the protein kinase Akt/PKB contributes to oncogenic transformation of cells [36]. Likewise, V599E and V600E mutations in BRAF kinase are associated with various carcinomas while BRAF somatic missense mutations occur in 66% of malignant melanomas [37]. The oncogenic mutations in JAK2 kinase such as single point mutation (Val617Phe) and JAK2 exon 12 mutations are implicated in both myeloproliferative disorders and myelodysplastic syndromes [38, 39]. Similarly, genetic alterations in other kinases such as ALK, IGF-1R, c-Kit, FGFR1–4, c-Met, c-Ret, c-SRC, regulate fundamental molecular mechanisms for tumor cell growth and development [9, 40]. Apart from tumor initiation, kinases are also vital for tumor cell survival and proliferation and may be present as downstream members of oncogenic kinase pathways. This category of kinases includes EGFR, a receptor tyrosine kinase, which has been shown to prevent autophagic cell death by maintaining intracellular glucose levels through interaction and stabilization of the sodium/glucose cotransporter 1 (SGLT1) [41]. Oncogenic alterations in EGFR make up approximately 45% of mutations in the tyrosine kinase domain [42, 43]. This leads to the loss of the inhibitory regulatory domains for dimerization resulting in hyper-proliferation of cancer cells via G1/S cell cycle progression [44, 45]. Other crucial members of the kinase family are aurora kinases (Aurora A-C). Aurora kinases are strategic kinases involved in defective spindle pole organization, and their pathophysiology correlates strongly with their oncogenic functions [46]. Aurora-A is an oncogenic kinase, and its amplification is documented in 10–25% of ovarian cancers [47]. Interestingly, Aurora A gene was originally named BTAK (breast tumor activated kinase) because its mRNA is overexpressed in breast cancer and is involved in the oncogenic transformation of breast cells [48]. Aurora A phosphorylates p53 at Ser215 and inhibits p53-DNA binding, disrupting cell cycle check activities [49]. It is also related to the activation of NF-κB, which boosts cancer cell survival by evading apoptosis [50]. Similar to Aurora-A, Aurora B and C are overexpressed in tumor cells and help cell survival, metastasis, and avoidance of apoptosis [51–53]. Other examples of tumor cell survival kinases include MEK1 [54], MEK2 [54], mTOR [55], and S6 kinase [56] which are all downstream members of MAPK, PI3K–Akt and EGFR pathway, respectively. In recent years, the mechanistic basis for developing kinase inhibitors from the second class of kinases has improved significantly. Types of serine/threonine kinases include MAP kinases (activated by protein phosphatases), ERK and stress-activated JNK and p38. Currently, there are about 30 Aurora kinase inhibitors in different stages of pre-clinical and clinical development [57]. The third category of kinases implicated in oncogenesis includes kinases overexpressed in tumors and surrounding tissues of cancers, which are important for the maintenance of tumors in the host. These include mutations in neurotrophic growth factor receptor which are involved in pilocytic astrocytoma, the most common childhood brain tumor [58]. Other examples include VEGFRs, fibroblast growth factor receptor (FGFR) kinases, protein kinase CK2 and TrkB [9, 16]. Overall, oncogenic kinases underlie and define multiple features of cancer including rapid proliferation, survival, growth, and metastasis, and have promoted the development of a plethora of kinase inhibitors. The fourth category of kinases, RTK with 58 known members and 20 subfamilies, were discovered more than a quarter of a century ago [40]. These kinases have a similar molecular architecture, and their mutations and aberrant activation are associated with carcinogenesis and angiogenesis. Four principal mechanisms are involved in abnormal RTK activation in human cancers; these include autocrine activation, chromosomal translocations, RTK overexpression, and gain-of-function mutations. RTKs are activated by growth factor binding by inducing receptor dimerization or in some cases subset of RTKs forms oligomers even in the absence of activating ligand [59, 60]. Principal members of RTK include 20 members including EGFR and others [61]. EGFR represent RTKs family as the well-studied kinase, implicated in several human cancers including lung cancer [62], glioblastoma [63], breast cancer [64], cervical carcinoma [65] and related mutations [66]. Several small-molecule inhibitors and monoclonal antibodies have been approved by FDA against RTKs for cancer therapy. The key drugs include Imatinib (against PDGFR, KIT, Abl, Arg), Sorafenib (against Raf, VEGFR, PDGFR, Flt3, KIT) and Lapatinib (against EGFR, ErbB2).

## Kinase discovery and development timeline

**Fig. 3:**
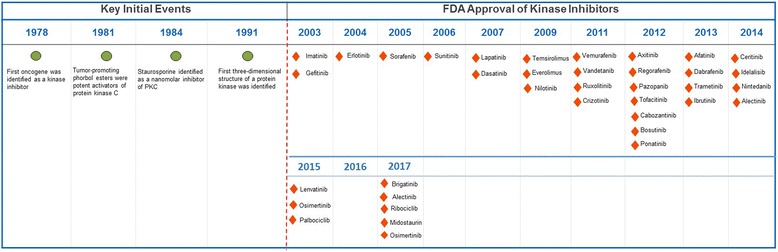
Timeline of key events in the development of protein-kinase inhibitors for the treatment of cancer

**Fig. 4:**
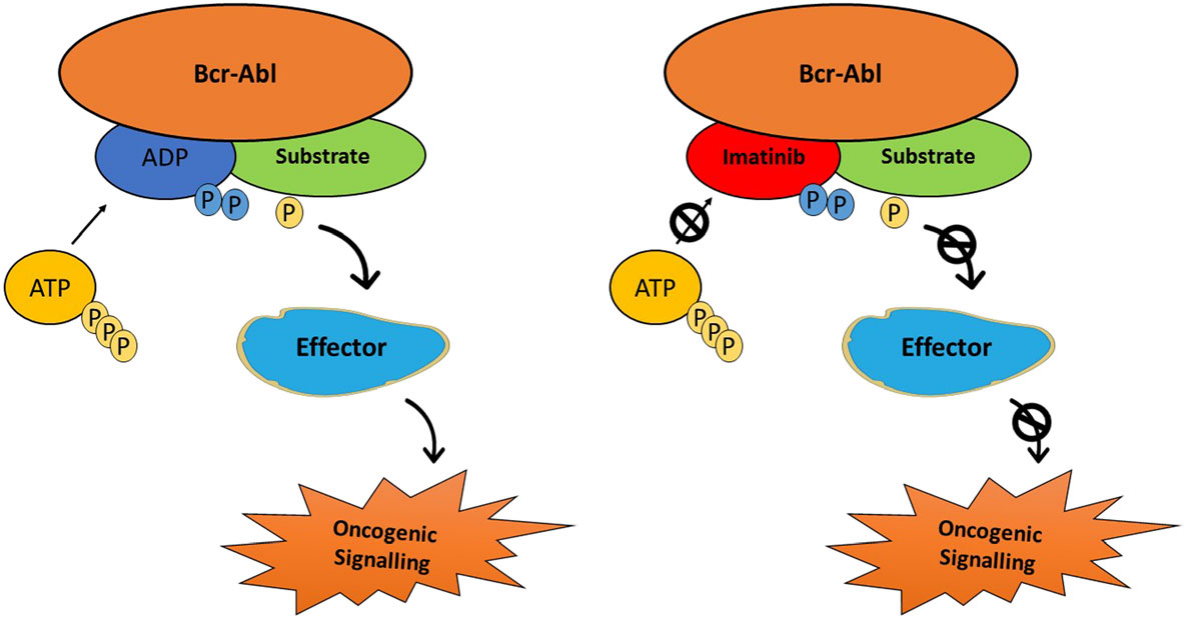
Interruption of the BCR-Abl pathway can be achieved by Gleevec (imatinib mesylate)

The development of kinase inhibitors for the treatment of human cancers started in mid 1970s (Fig. 3). In 1978, the first oncogene was found to be a protein kinase [67]. This discovery was supported by a successive finding in 1981 when tumor-promoting phorbol esters was shown to exhibit hyperactivation of protein kinase C (PKC) [68]. In the coming years, naphthalene sulphonamides, the first protein kinase inhibitors were synthesized and served as a basic design for developing further molecules [69]. During this time, staurosporine, an antifungal drug was shown to be a nanomolar inhibitor of PKC [70]. This drug was later used as a parent compound to produce various analogs as potential inhibitors of PKC. In the 1991 the 3-D structure of protein kinase A (PKA) was elucidated, and it became apparent that the residues that were involved in binding ATP were conserved from kinase to kinase [71, 72]. This discovery perpetuated a myth that it was “impossible” to develop protein-kinase inhibitors with the requisite potency and specificity. However, with the discovery of cellular targets for cyclosporin and subsequent development of HA1077, an inhibitor of several protein kinases, the field of kinase inhibitors rapidly progressed [14, 73, 74]. Finally, the breakthrough occurred in 2001 when imatinib, a phenyl-amino-pyrimidine derivative targeting the inactive conformation of the ABL1 kinase, was approved for the treatment of CML (Fig. 4). Starting with a 2-phenylaminopyrimidine derivative, chemists added a 3′ pyridyl group, benzamide, a flag methyl instead of N-methylpiperazine to synthesize a drug candidate called CGP57148B (later changed to imatinib) [75]. Clinical targeting of BCR-ABL gene, formed by the fusion of ABL gene from chromosome 9 to the BCR gene on chromosome 22, also called the Philadelphia chromosome, improved the clinical management of leukemia patients [76, 77]. Owing to its’ broad-spectrum nature imatinib has since then been approved for various other oncology indications. Following the FDA approval of imatinib, different strategies have been used for the development of single and multi-target kinase inhibitors for cancer treatment [78]. More active drugs, such as nilotinib, with a selectivity profile similar to imatinib, were approved for imatinib-resistant CML [79, 80]. Later on, the indolinone-derivative sunitinib with a broad spectrum activity targeting VEGFR, PDGFR, FGFR, KIT, and FLT3, was approved for the treatment of renal cell carcinoma, as well as second-line therapy in the imatinib-resistant gastrointestinal stromal tumor (GIST) [81]. Sorafenib was later approved for the treatment of renal cell and hepatocellular carcinoma due to its ability to bind to the inactive conformation of the VEGFR kinase [82]. Similarly, in the year 2009, pazopanib, a 2-amino pyrimidine targeting VEGFR, PDGFR, and KIT was approved for the treatment of advanced renal cell carcinoma [83]. A quick surge in clinical approval of kinase inhibitors started following the approval of everolimus (mTOR inhibitor) in 2009 for the treatment of metastatic renal cell carcinoma (mRCC), astrocytoma and breast tumors [84–86]. In the year 2011, four kinase inhibitors, vemurafenib, vandetanib, ruxolitinib, and crizotinib were approved for the treatment of melanoma, thyroid cancer, myelofibrosis and ALK-positive non-small cell lung cancer [87–90]. Successively, in 2012 and 2013 ten new kinase inhibitors were approved by FDA for the treatment of various malignancies. Since the initial development of imatinib, 28 kinase inhibitors have been approved by FDA with Brigatinib and Osimertinib being the latest approvals [91, 92]. Apart from the approved kinase inhibitors, there is more than three thousand ongoing Phase I-III clinical trials for hundreds of new kinase inhibitors. It is therefore beyond the scope of this mini-review to discuss all the protein kinase inhibitors that are in clinical Phase I–III.

## Types of kinase inhibitors

Kinase inhibitors are very efficacious for the treatment of cancer especially targeting specific mutations that chiefly drive tumorigenesis. They are categorized according to their capacity to catalyze the transfer of the terminal phosphate of ATP to the substrates that usually contain a serine, threonine or tyrosine residue (Table 2). Many reviewers have categorized types of kinase inhibitors according to their mechanism of action. Initially, small molecule protein kinase inhibitors were divided into three classes, termed as types I, II, and III kinase inhibitors [93]. Dar and Sakot defined the type I kinase inhibitor as “a small molecule that binds to the active conformation of a kinase in the ATP pocket,” the type II inhibitor as “a small molecule that binds to an inactive (usually Asp-Phe-Gly (DFG)-OUT) confirmation of a kinase,” and the type III inhibitor as “a non-ATP competitive inhibitor” or allosteric inhibitor [93, 94]. Later on, Zuccotto et al. introduced a new class of kinase inhibitors, i.e. type I½ inhibitors, which bind to the protein kinases with the DFG-Asp in and C-helix out conformation [95]. Later, Gavrin and Saiah further divided the allosteric effectors into two subclasses (III and IV) where the type III inhibitors bind within the cleft between the small and large lobes adjacent to the ATP binding pocket and type IV inhibitors bind outside of the cleft and the phosphor-acceptor region [96]. Afterwards, bivalent molecules that span two regions of the protein kinase domain were labeled as type V inhibitors [97]. Finally, small molecules that form covalent adducts with the target enzyme were recently termed as covalent inhibitors [94]. The classification described herein uses these parameters with added subdivisions and criteria, labeling them as types I, II, allosteric, and substrate directed and covalent inhibitors.

**Table 2:** Classification of small molecule kinase inhibitors

Class of Kinase Inhibitor	Mechanism of Action	Examples
Type I	Competes for the substrate and binds in the ATP-binding pocket of the active conformation	Bosutinib, Cabozantinib, Ceritinib, Crizotinib, Gefitinib, Pazopanib, Ruxolitinib, Vandetanib
Type II	Type II inhibitors bind to the DFG-Asp out protein kinase conformation, which corresponds to an inactive enzyme form	Imatinib, Sorafenib, Axitinib, Nilotinib
Type III (Allosteric Inhibitor)	Occupy a site next to the ATP-binding pocket so that both ATP and the allosteric inhibitor can bind simultaneously to the protein.	Trametinib, GnF2
Type IV (Substrate Directed Inhibitors)	Undergo a reversible interaction outside the ATP pocket and offer selectivity against targeted kinases	ONO12380
Type V (Covalent Inhibitor)	Bind covalently (irreversible)to their protein kinase target	Afatinib, Ibrutinib, HK1–272

### Type I kinase inhibitors

Type I kinase inhibitors represent ATP-competitors that mimic the purine ring of the adenine moiety of ATP. Functionally, they interact with the conformational phosphorylated active catalytic site of the kinases. These kinase inhibitors bind to the active conformational site and alter the structural conformation otherwise favorable to phosphotransfer [98, 99]. Type I inhibitors usually contain a heterocyclic ring system that occupies the purine binding site, where it serves as a scaffold for side chains that occupy adjacent hydrophobic regions [100]. These hydrophilic regions of the enzyme occupied by the ribose moiety of ATP may be used to exploit the solubility of the drugs or other active compounds [98]. To date, many Type I kinase inhibitors for the treatment of cancer have been approved by the FDA viz. bosutinib, crizotinib, dasatinib, erlotinib, gefitinib, lapatinib, pazopanib, ruxolitinib, sunitinib, and vemurafenib. Apart from the large-scale clinical success, Type I kinase inhibitors also come with adverse side-effects. Type I inhibitors display an inclination for low kinase selectivity as the targeted ATP pocket is conserved through the kinome; therefore, increasing the potential for off-target side effects. This little selectivity for targeted kinases may result in cardiotoxicity and possible deterioration in cardiac function [101, 102].

### Type II kinase inhibitors

Type II kinase inhibitors act by targeting the inactive conformation of kinases and interact with the catalytic site of the unphosphorylated inactive conformation of kinases [103]. Type II kinase inhibitors exploit new interactions inside the lipophilic pocket derived from the change of confirmation of the phenylalanine residue of the “Asp-Phe-Gly (DFG)” *N*-terminal loop conformation of kinases [16, 103]. These inhibitors interact reversibly with the target kinase which leads to the formation of single or multiple hydrogen bonds with the protein in the ‘hinge region’ and also causes extra interactions in the open DFG-out conformation [98, 103]. These lipophilic interactions have a high degree of selectivity towards unwanted kinases affecting an increase in the safety profile of Type II kinase inhibitors. Type II inhibitors also display a high conservation of distinctive H-bond pattern between the inhibitor and the glutamic and aspartic acids of the kinase [98, 104]. Due to the exclusivity of inactive protein kinase conformations, it was theorized than type II kinase inhibitors would be more selective. However, there is considerable overlap of selectivity between type I and type II inhibitors. The discovery of Type II kinase inhibitors such as imatinib and sorafenib was serendipitous, and it wasn’t until much later that their mode of action was discovered. The role of imatinib in the consequent development of small molecule protein kinase inhibitors cannot be overstated. All Type II inhibitors share a similar pharmacophore and hydrogen bonds that interact with DFG-out kinase conformational structure as revealed by the discovery of the Type II kinase inhibitor co-crystal structure [105]. Since canonical ATP-binding sites of activated kinases, the target sites of Type I inhibitors, do not share these features, this pocket is conserved to a lesser extent across the kinome, and hence promises better prospects for the rational design of selective inhibitors [100, 103]. Overall, Type II kinase inhibitors display high selectivity towards kinase inhibition as compared to Type I kinase inhibitors along with the profound impact on cellular activity.

### Type III or allosteric inhibitors

The third class of kinase inhibitors bind outside the catalytic domain/ATP-binding site and modulates kinase activity in an allosteric manner. Some authors have divided the allosteric inhibitors into two subtypes where type A inhibitors bind to an allosteric site next to the adenine-binding pocket whereas the type B inhibitors bind elsewhere [97]. Overall, Allosteric or Type III inhibitors exhibit the highest degree of target kinase selectivity as they exploit binding sites and physiological mechanisms that are exclusive to a particular kinase [106]. With respect to ATP, these drugs are steady-state noncompetitive or uncompetitive inhibitors because ATP cannot prevent their interaction with the target kinase. One of the earliest allosteric inhibitors was CI-1040, an orally active, highly specific, small-molecule inhibitor of the MEK1/MEK2 pathway [107]. A recent chemical proteomics study confirms the allosteric activity of type III inhibitors as they showed a higher selectivity, but also stated that these are special cases as most of them are designated MEK1/2 inhibitors that bind to a particular cavity adjacent to the ATP-binding site [108]. Another allosteric kinase inhibitor GnF2 binds to the myristate binding site of BCR–ABL1 [109]. GnF2 also displays sound IL-3 reversible anti-proliferative and apoptotic effect on two mutants identified as E255V and Y253H [109]. Likewise, TAK-733 binds to the MEK1-ATP complex in the gate area and the back cleft adjacent to the ATP-binding pocket; however, it cannot bind to the adenine pocket owing to its occupation by ATP [110]. Other examples include RO0281675 and analogs thereof [111, 112]. Overall, targeting kinases using allosteric inhibitors is thought to be a crucial approach for overcoming hurdles in kinase inhibitor research, such as limited selectivity, off-target side effects, and drug resistance. In future, more active and target specific allosteric inhibitors will be discovered as larger stress is placed on cell-based assays in which kinases are explored in their native cellular context.

### Substrate-directed inhibitors

These are also called Type IV kinase inhibitors and undergo a reversible interaction outside the ATP pocket, located in the kinase substrate-binding site. These inhibitors don’t compete with ATP and offer a higher degree of selectivity against targeted kinases [113]. Substrate-directed inhibitors include ATP-noncompetitive inhibitors such as ON012380 which are targeted against Philadelphia chromosome-positive leukemias [114]. More importantly, ON012380 was found to override imatinib resistance at physiologically relevant concentrations of < 10 nM [115].

### Type V or covalent inhibitors

The covalent kinase inhibitors form an irreversible covalent bond with the kinase active site and target a catalytic nucleophile cysteine within the active site of the enzyme [116, 117]. The chemical rationale for developing Type V inhibitors is based on exposed cysteine side chain in the ATP site which can be targeted for covalent reaction with a drug candidate with an electrophilic Michael acceptor in the right position [118, 119]. This type of kinase inhibition takes place via trapping of a solvent-exposed cysteine residue either by S_N_2 displacement of a leaving group or by reacting with a Michael acceptor incorporated within the kinase inhibitor [113, 120, 121]. Covalent inhibitors target respective kinase by formation of a rapidly reversible collision complex followed by an irreversible enzyme-inhibitor complex [122]. Afatinib (targets EGFR (ErbB1), ErbB2, and ErbB4) and ibrutinib are currently FDA-approved drugs that form a covalent bond with their target kinase. Afatinib, unlike the first-generation EGFR-TKIs such as gefitinib and erlotinib, is a mutant-selective EGFR inhibitor with low toxicity profile despite its irreversible mechanism [123]. Similar to Afatinib, ibrutinib also targets mutant-EGFR kinase with a distinct binding conformation [124]. Both of these kinase inhibitors initiate Michael reaction with the addition of a nucleophile (the -SH of cysteine) to an α, β unsaturated carbonyl compound [125]. C481 within hinge region of the Bruton tyrosine-protein kinase is hypothesized to form a covalent link with ibrutinib [126]. A recently approved kinase inhibitor, neratinib (HKI-272), inhibits Herceptin-2 (HER-2), and prevents recurrence in patients with early-stage HER2-positive breast cancer [127]. Overexpression of HER-2 is seen in 25–30% of breast cancer patients and predicts a poor outcome in patients with primary disease. Likewise, CL-387785, a covalent inhibitor, overcomes resistance caused by T790 M mutation of the epidermal growth factor receptor (EGFR) [128]. These kinase inhibitors also display an extended dissociation half-life which minimizes off-target side effects [118]. Other advantages include prolonged pharmacodynamics, suitability for rational design, high potency, and ability to validate pharmacological specificity through mutation of the reactive cysteine residue [119]. The approved covalent kinase inhibitors (Ibrutinib, Afatinib, and Neratinib) have shown that small molecules containing weak reactive electrophiles can be mutant specific in action with low toxicity. These kinase inhibitors have initiated resurgence of interest in covalent inhibitors, and feature an acrylamide functionality to specifically target the cysteine side chains of kinases. Example include a recent study showing nine irreversible EGFR and two BTK inhibitors with higher kinase inhibitory selectivity than reversible compounds [108]. The Type V or covalent kinase inhibitors have substantial potential for exploration as 200 different kinases have a cysteine chain located near the ATP pocket.

## Biochemical mechanism

Biochemically, kinase inhibitors are classified according to the activation state of the protein kinase target including the nature of DFG-Asp (active in, inactive out), the C-helix (active in, inactive out), and the regulatory spine (active linear, inactive distorted). Apart from type III or allosteric inhibitors, all the FDA-approved kinase inhibitors form hydrogen bonds with one or more hinge residues. Overall, most kinase inhibitors form: (i) hydrophobic contacts with catalytic spine residues; (ii) contact with the RS3 R-spine residue within the C-helix; (iii) interaction with the gatekeeper residue; and (iv) residues that occur just before the DFG-D of the activation segment [94, 129]. The following section briefly discusses the biochemical mechanism of action of FDA-approved kinase inhibitors.

Frequent mutations in various protein kinases present specific kinase inhibition as a therapeutically relevant approach in oncology. Kinase inhibitors have evolved to target many different regulatory and inhibitory mechanisms. There are various mechanisms by which kinase inhibitors bind to their target kinases broadly classified into kinase inhibitors that bind either covalently or non-covalently to or around the ATP binding site. Primarily, kinases bind with ATP in a cleft between the N- and C-terminal lobes of the kinase domain. In this domain, the adenine group of ATP is bound by two hydrophobic surfaces and interact via hydrogen bonds to the connector of two lobes, called the “hinge region” [130–132]. The cleft of ATP contains various elements such as the flexible activation loop (A-loop), along with closed conformations which are responsible for the catalytic activity of the kinase [133, 134]. The active or inactive state of the protein kinase is determined by the position of the A-loop, including the DFG motif at its N-terminal, which has various conformations [28, 98, 134, 135]. The only component of kinases that does not vary between the active and inactive states is the catalytic loop. The active state of the protein kinase when the Asp in the DFG motif coordinates one magnesium ion, which prepares the phosphates of ATP for the transfer of the phosphoryl group. The Phe in the DFG motif packs under the helix-C positioning both helix-C and A-loop for catalysis [98, 133, 136]. Protein kinases return to their inactive conformation once kinase transfers the phosphoryl group from ATP to tyrosine, serine or threonine of the substrate protein. This process also involves the returning of the A-loop to the closed position by the change of A-loop from the DFG-in to the DFG-out conformation [98, 137, 138]. However, ribose binding and the phosphate binding site of ATP usually remains unexplored by the majority of kinase inhibitors [134, 139]. Based on the biochemical mechanisms of action, kinase inhibitors are categorized as covalent and non-covalent kinase inhibitors. The non-covalent kinase inhibitors are classified into those who either bind or do not bind to the hinge region of the kinase [140]. The DFG-in or Type I kinase inhibitors bind to hinge region and represent the vast majority of non-covalent kinase inhibitors [98]. In these kinase inhibitors, the Asp in the DFG motif coordinates the phosphates of ATP, and the Phe in the DFG motif stabilizes the position of helix-C and the A-loop for catalysis [20]. However, the ATP-binding pocket is highly preserved among members of the kinase family, and it is hard to find highly selective Type I kinase inhibitors. Moreover, the pre-clinical to clinical translation of Type I kinase inhibitors is hindered as they compete with high levels of intracellular ATP leading to a discrepancy between biochemical and cellular analysis. Contrary to the Type I inhibitors, Type II inhibitors bind to the DFG-out confirmation of kinases. These inhibitors induce a conformational shift in the target enzyme such that the target kinase is no longer able to function. Type II inhibitors use an additional hydrophobic pocket adjacent to the ATP site exposed by the movement of A-loop from DFG-in to DFG-out conformation [141]. This gives the Type II inhibitors higher selectivity as they recognize novel regions of the active cleft outside the highly conserved ATP-binding site. Like Type II kinase inhibitors, the allosteric inhibitors or Type III inhibitors also display high selectivity as they explore binding sites and regulatory mechanisms that are unique to a particular kinase. They contain a heterocyclic system that forms one or two hydrogen bonds with the kinase hinge residue. Like Type II inhibitors, they also induce the DFG-out confirmation and move phenylalanine side chain to a new position [98, 99]. Examples include compounds such as CI-1040, which inhibit MEK kinase by occupying a pocket adjacent to the ATP-binding site [107]. Interestingly, exploration of allosteric kinase inhibitors also helps to recognize unique kinase activation targets, which could be explored for therapeutic intervention in other diseases states. Recently, there has been an increased interest in the development of irreversible (covalent) kinase inhibitors that form covalent bonds with cysteine or other nucleophilic residues in the ATP-binding pocket. These inhibitors have typically been developed by incorporation of an electrophilic moiety into an inhibitor that already possesses submicromolar binding affinity to the target of interest. The covalent kinase inhibitors bind to a cysteine residue in or around the active site, thus preventing the binding of ATP to the protein kinase [117, 127]. These kinase inhibitors undergo the “Michael reaction”, which is a reaction that triggers the addition of a nucleophile, such as a cysteine, to an α, β unsaturated carbonyl functionality. Nucleophile additions cause the formation of adducts at the electrophilic β-position and inactivate kinases by irreversibly blocking the binding of ATP to kinase [142]. These kinase inhibitors are highly selective as they overcome endogenous ATP competition and target a specific cysteine at the corresponding position in a kinase. Various covalent kinase inhibitors target kinases such as BTK [143], Fes [144], VEGF-R2 [145], and RSK2 [146] through their ability to bind to a cysteine residue.

## Recent clinical developments

Traditional cancer therapies follow palliative as well as off-targeted approaches in oncology. In contrast, kinase inhibitors symbolize a class of targeted cancer therapeutic agents with limited nonspecific toxicities. So far, 28 inhibitors with activity targeted to one or multiple kinases have been approved for clinical use. With over 500 members, the kinase family has received a high degree of attention from academic researchers as well as pharmaceutical industries [147]. After the clearance of possible hindrances, owing to the high degree of active site similarities and possible off-target activity, kinase inhibitors have gained scientific limelight [21, 24, 78, 148, 149]. In a 13-year summary of targeted therapies including kinase inhibitors, the clinical success rate of kinase inhibitors was superior to other cancer therapies [150, 151]. Nevertheless, this clinical success does come with exceptions; attempts to control cytotoxicity during treatment, particularly with sunitinib and EGFR/VEGF-system targeting drugs have yielded disappointing results [152–155]. Overall, during the last 5 years, Aurora kinases [156], casein kinase II [157], cyclin-dependent kinases [158], focal adhesion kinase [159], protein kinase B [160], phosphatidylinositol 4,5-bisphosphate 3-kinase delta and gamma [161], polo-like kinase I [162], tyrosine-protein kinase SYK [163], high affinity nerve growth factor receptor family [164] and Wee1-like protein kinase [165] have been targeted in Phase I clinical trials. Although recent developments have shown Aurora kinases as major new targets in kinase inhibitor development [166, 167]. After initial hurdles, two compounds palbociclib and ribociclib have passed the phase III clinical trials and are in clinical use [168].

Recent kinase developments include precision therapy based on tumor genomic data. The ability to perform genetic studies of tumors and follow-up treatment decisions based on the identification of tumorigenesis drivers has resulted in significant benefits for patients in need of effective systemic therapy. The detailed information regarding all the clinical trials is out of the scope of this mini-review; however, a few important developments are highlighted. A small number of small molecule tyrosine kinase inhibitors have recently received FDA approval for treatment of non-small cell lung cancer (NSCLC) with EGFR mutations or ALK translocations [169]. Afatinib, a second-generation, non-competitive kinase inhibitor targeting all members of the ErbB family of receptors (also known as Her-2/neu) was approved in 2013 as frontline therapy for NSCLC patients with EGFR-deletion 19 and L858R mutations [170]. Despite several challenges that need to be overcome, reviewed in [171, 172], precision medicine has yielded important dividends for patients with advanced cancers [173]. In order to counter currently undruggable targets and acquired resistance, immunotherapy has gained widespread recognition in recent years [174]. Additionally, kinase targeted antibody therapy for hematological malignancies, and solid tumors have become established over the past 20 years. Key examples of antibody constructs targeting kinases include Trastuzumab and T-DM1 (targeting ERBB2/HER2) in breast and bladder cancer [175, 176], Bevacizumab (targeting VEGF) in ovarian, metastatic colon cancer and glioblastoma [177], Cetuximab, Panitumumab and necitumumab (targeting EGFR) in colorectal cancer and NSCLC [178]. Other experimental candidates include scFv, affibody and minibody (ERBB2/HER2 and FGFR1) [179–182], Protein–Fc (VEGFR1 and VEGFR2) [183] and Intact IgG (EGFR, ERBB2, and VEGF) in breast and lung cancer studies. Also, there is an increased development of PI3K and mTOR inhibiting compounds. Dual PI3K/mTOR inhibitors in advanced clinical trials include NVP-BEZ235 (glioblastomas) [184], XL765 (breast cancer) [185], GDC0980 (mRCC) [186], PF04691502 (breast cancer) [187], GSK2126458 (colorectal, breast, non-small cell lung, and pancreatic cancers) [188], Quinacrine (various leukemias) [189, 190] and PKI587 (advanced solid malignancies) [191]. Also, buparlisib and idelalisib, both PI3K inhibitors, have entered phase III clinical trials [192, 193]. In line with PI3K/mTOR inhibitors, various kinase inhibitors have entered into clinical trials for gastrointestinal cancers [194], thyroid carcinoma [195], breast cancer [196], and endocrine tumors [197]. Many previously approved kinase inhibitors are being tested in clinical trials against BRAF and cyclin-dependent kinases 4/6 mutations [20, 99]. BRAF somatic mutation, particularly BRAF V600E/K, drive tumorigenesis through constitutive activation of the downstream MAPK pathway [198]. Multiple drugs including vemurafenib, dabrafenib, PLX3603, ARQ736, CEP-32496, BMS-908662, BGB283, encorafenib in combination with other chemotherapies are being targeted for BRAF-mutated cancers [199]. It is now suggested that dabrafenib, a selective BRAF inhibitor may target other kinases indicating polypharmacology (that is, drugs that act on more than one target) [108]. A paper published by Klaeger and colleagues explains the potential of 243 clinically evaluated kinase drugs [108]. Although multiple new kinases have been targeted during the last 5 years, a large share of the cancer kinome is still untargeted. Furthermore, use of these targeted therapies is not without limitations. Reservations on the use of kinase inhibitors include the development of resistance and the lack of tumor response in the general population and these constraints still need to be resolved.

## Natural bioactives as kinase inhibitors

**Fig. 5:**
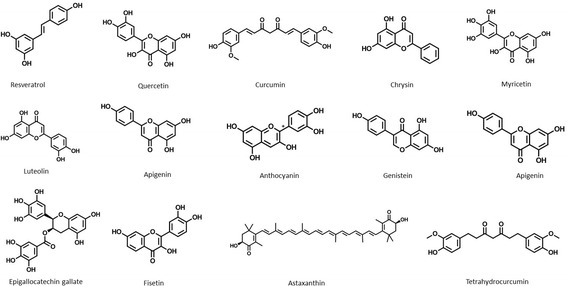
Structures of key natural bioactives which pharmacologically modulate kinases

Overexpression of kinases is observed in multiple carcinomas. In recent years, there has been a major paradigm shift in discovery and screening of natural compounds as potential kinase inhibitors. Emerging data has revealed numerous mechanisms by which natural compounds mitigate kinase mutations. Classically, many of the biological actions of small molecule compounds, especially polyphenols, have been credited with their antioxidant properties, either through their reducing capacities or their possible influence on intracellular redox states. These small molecule bioactives can directly bind receptor tyrosine kinases and alter their phosphorylation state to regulate multiple cell signaling pathways (Fig. 5). Elevated levels of the EGFR and HER-2 have been identified as common components of multiple cancer types and appear to promote solid tumor growth [200, 201]. EGFR inhibition is exhibited by multiple polyphenols including resveratrol [202], quercetin [203], curcumin [204], and green tea extracts [205]. HER-2 overexpression in tumor cells is also attenuated by these bioactives [206–208]. Fibroblast growth factors are involved in a variety of cellular processes, such as tumor cell proliferation, drug resistance, and angiogenesis [209]. Oncogenic alterations of RTK kinases including FGFR1, FGFR3, and FGFR4 are inhibited by natural compounds [210–212]. Similarly, curcumin and chrysin block expression of receptor d’origine nantais (RON) in tumor cells [213, 214]. The product of the human SRC gene, c-Src, is found to be over-expressed and highly activated in a wide variety of human cancers [215]. It is also accompanied by elevated levels of Abl [216] and JAK-2 kinases [217]. Interestingly, the overexpression and translocation of oncogenic cytoplasmic tyrosine kinases such as c-SRC [218], Abl [219], c-Met [220] and JAK-2 [221, 222] are tempered by natural compounds. Serine/threonine kinases, within the kinase family, play vital roles regarding their involvement in human cancers. Akt, a crucial kinase modulates diverse cellular processes involved in the regulation of cell survival, cell cycle progression and cellular growth [223]. Up to date, more than 50 proteins have been identified as the phosphorylation substrates of Akt. Resveratrol modulates expression of Akt in breast [224], uterine [225], prostate [226, 227], skin [228] and glioma cells [229]. It targets the kinases at ATP-binding site competitively and reversibly [230, 231].

Furthermore, myricetin has been reported to target Akt to inhibit cell transformation and proliferation by directly binding to the ATP-binding site of Akt [232]. Similar effects are also exhibited by curcumin [233], quercetin [234, 235], green tea molecules [236], anthocyanins [237] and other polyphenols [238–240]. Hyperactivity of cyclin-dependent kinases (CDKs) is one of the key mechanisms underlying tumorigenesis [241]. The overexpression of CDKs is inhibited by various small molecule compounds [242–246]. Likewise, hyperactivity of mTOR pathway is also downregulated by natural compounds [229, 247–249]. The mTOR pathway is a critical effector in cell-signaling pathways and is commonly deregulated in human cancers. Furthermore, small molecule compounds also inhibit the activity of polo-like and Aurora kinases [207, 210, 250, 251]. B-Raf kinases, key kinases intimately involved in cancer cell proliferation [252], are also inhibited by natural plant compounds such as curcumin, luteolin, quercetin and ursolic acid [253, 254]. Finally, the overexpression of oncogenic lipid kinases such as PI3K and SK1 is also mitigated by small molecule bioactives. More than 30% of various solid tumor types were recently found to contain mutations in PI3K [255]. Well explored bioactive molecules such as resveratrol [228], curcumin [256], quercetin [235] and green tea polyphenols [257] inhibit PI3K pathway. Similar to the parent compounds, metabolites of bioactives also inhibit PI3K pathway [258]. Sphingosine kinase 1 (SphK1) is also an important component of carcinogenesis as it converts the proapoptotic lipids ceramide and sphingosine into the anti-apoptotic lipid sphingosine-1-phosphate [259]. Inhibition of SphK1 is exhibited by few chelating bioactives [260–262]. Oncogenic kinases are vital proteins that couple extracellular signals with intracellular signaling pathways, which contribute to all stages of cancer development. Accumulated data reveals that plant compounds, particularly polyphenols, exert anti-cancer effects through acting on protein kinase signaling pathways. Many natural bioactives bind directly to oncogenic protein kinases and then alter their phosphorylation state, thus mitigating cell signaling pathways in carcinogenesis processes.

## Challenges and limitations

Despite numerous advances, scientists are still trying to understand pathophysiology and application of kinase inhibitors for therapeutic benefit in clinical oncology. Kinase inhibition triggers a strong discerning pressure for cells to acquire resistance to chemotherapy through kinase mutations [263]. Thus, the treatment and pathology of cancer are further complicated by the plethora of such mutations that occur in different kinases [264]. There are two types of chemotherapy resistance: de novo resistance, which refers to the failure of an agent to produce any detectable response after initial treatment and acquired resistance. Multiple mechanisms including the targeted kinase, the structure of inhibitor, and the underlying genetic features of the tumor contribute to treatment failure and both types of resistance. Acquired resistance refers to the progression of a tumor that initially responds to treatment and subsequently becomes resistant to treatment despite continual administration of the inhibitor. Interestingly, most of the kinase resistant cases fall into the acquired resistant category. Treatment resistance associated with kinase inhibitors is induced by changes in the kinase gatekeeper residue as hydrophobic interactions on this site are crucial for the binding affinity of the inhibitor [265, 266]. Since a small gatekeeper residue allows an inhibitor to access the “gated” hydrophobic regions of the binding pocket, changes in this region hinder activity of kinase inhibitors. The gatekeeper residue has no interaction with ATP but is usually in contact with Type I and Type II kinase inhibitors and sterically impedes inhibitor binding [98]. These mutations mainly lead to in the substitution of one amino acid for another in the protein made by a gene, thus conferring resistance to cell cycle termination and chemo drugs. A classic example is induction of imatinib resistance due to gatekeeper mutations in Thr 315 (coded by ACT) in BCR-ABL kinase [254]. Other examples of such gatekeeper mutations include T790 (EGFR) [267], G697R (FLT3) [268], BCR–ABL1 (T315I) [269], PDGFRα (T674I) [270] and KIT (T670I) [271] oncogenic mutations. In the case of the EGFR kinase, the T790 M mutation induces resistance to quinazoline inhibitors by increasing affinity for the natural substrate ATP [272]. It is one of the most common mutations in which methionine substitutes for threonine at amino acid position 790, conferring a growth advantage to cancer cells alongside drug-resistant variant of the targeted kinase [273]. Similarly, 20% of cases of acquired TKI resistance involve amplification of the MET gene [274]. These events thereby provide signalling redundancy and eliminate consequences of clinical kinase inactivation. Furthermore, the lipid modifying PI3K together with the Ras-Raf-MAPK also undergoes several resistance-inducing mutations [275]. Interestingly, these mutations cause a minute or no change in kinase activity but confer inhibitor resistance to kinase inhibitors [276]. An example includes gatekeeper mutation T790 M in EGFR which causes gefitinib and erlotinib resistance via hyper affinity for ATP [277, 278]. Overcoming gatekeeper-mutation induced drug-resistance in the clinic is extremely difficult and requires structural fine-tuning of the drug candidates. To surmount resistance to inhibitors gefitinib and erlotinib, kinase inhibitors that bind covalently to the ATP-binding site of EGFR are been developed [117, 279]. Such next-generation EGFR inhibitors selectively target the inhibitor-sensitizing mutations and display an improved safety profile by sparing wild-type EGFR activity in normal cells. A recent study using chemical proteomics analyzed 243 clinically evaluated kinase drugs and showed that some kinase inhibitors are highly selective, especially KIs targeting mutant EGFR [108]. Likewise, G-loop mutations in ABL, p38α, FGFR1, CK2α1, JNK3, AURORA-A, ROCK1 and CDK5 kinases prompt oncogenic or drug-sensitizing mutations [280]. Another clinical challenge associated with kinase inhibitors is variation in clinical results from combinations of kinase inhibitors. Examples of clinical failure include combined gefitinib and trastuzumab treatment in breast cancer, erlotinib and bevacizumab in renal cell carcinoma, and cetuximab and bevacizumab in colorectal cancer. Conversely, combinations of lapatinib and pertuzumab with trastuzumab in breast cancer, and combination of bevacizumab and erlotinib in NSCLC have exhibited clinical success. Further, in some cases, the combinations of kinase targeting agents reduced patient survival compared with the treatment using single drug [281]. However, these discrepancies are proposed due to misinterpretation of the preclinical data, rather than a failure of the preclinical model itself [282, 283]. Additionally, these preclinical studies of drug combinations are probably biased towards validating well-characterized targets thereby limiting their ability to prioritize novel targets. Further, many kinase inhibitors are associated with toxicities and off-target effects such as cardiotoxicity, hypertension, hypothyroidism, skin reactions and proteinuria [284, 285]. Looking specifically, inhibition of EGFR is associated with dermatological problems, VEGFR inhibition with cardiotoxicity, HER2 and ALK inhibition with gastric irregularities and dermatological problems, and BCR-ABL inhibition causes cytopenia, in addition to cardiotoxicity and cardiac complications [286, 287]. Another challenge is in translating RNAi therapy into drugs, particularly in kinase inhibition. The majority of drug targets cannot be battered by shRNA (or gene knockout) as most shRNAs cannot be replicated by drugs since most proteins cannot be translated to therapy [288]. Thus, clinical resistance to kinase inhibitors remains the major limitation to kinase-based -therapies. Resistance to chemotherapy has also been well recognized as a significant challenge in oncology, a problem also confronted by kinase inhibitors. Beyond the stated illustrative examples, numerous other pathways outside the scope of this review can influence the clinical activity of kinase inhibitors.

Numerous follow-up strategies are being employed to overcome the challenge of kinase inhibitor resistance. A first approach is to develop inhibitors that can tolerate diverse amino acids at the gatekeeper position [289, 290]. A second approach is to target the kinase with inhibitors that bind at alternative binding sites [115, 291]. A third approach involves targeting other pathways that may be required for kinase transformation [292]. These approaches have been demonstrated to work in cell line studies, and strategies are being developed for their clinical use. However, it is also vital to consider the possibility that multiple different resistance mechanisms might develop concurrently in patients, thereby challenging clinical ability to overcome acquired resistance to kinase inhibitors.

## Future developments

Even though only a small fraction of the kinome is currently being targeted, kinase inhibitor drug discovery has progressed dramatically in the past decade. Clinical evaluation of kinase inhibitors has shown that therapeutic responses vary widely in individual patients and across patient populations, and seem to depend on many diverse factors. Many new candidate molecules have entered clinical trials, and much more are still at the preclinical stage. Most of the current kinase inhibitor discoveries have developed through rational drug design rather than through random screening and analysis of structure-activity relationships. An important strategy required for future development is to understand the basis of unexpected toxicities related to kinase inhibitors. Improvement in the documentation of toxicities of kinase inhibitor would provide a valuable database for understanding whether there are particular kinases of which inhibition should be avoided or specific substructures that result in problematic metabolites. This strategy will help to develop kinases with better selectivity benefitting the vast patient population. Also, there is a critical need for better ways to monitor target kinase inhibition in humans using minimally invasive techniques. This may include monitoring of cancer biomarkers that may serve as benchmarks for the clinical development of kinase inhibitors. The development of such technologies will help to discover and eradicate tumors using targeted kinase inhibition with minimal toxicities. There is also an urgent need for developing more non-ATP-competitive kinase inhibitors as the current collection of kinase inhibitors is limited to ABL, IKK, AKT, CHK1, MEK, SRC, IGF1R inhibitors [99, 293–296]. Furthermore, there is need to develop sophisticated modeling of chemotherapy resistance in response to kinase inhibitors. This will help to overcome kinase resistance and allow for the systematic application of combinations of kinase inhibitors. Furthermore, novel pre-clinical models are required to identify the best cocktails of kinase inhibitors combined with natural bioactives. Advanced high-throughput cell-based screening using well-defined phosphorylation readouts should be established. However, it may prove challenging to screen and develop natural kinase inhibitors using the cellular readout only. It is also important to understand that kinase inhibitors are not only important for the treatment of cancer, but also help us better understand the physiological roles of kinases. In the field of oncology, kinase inhibitors are proving to be well tolerated compared with conventional cytotoxic chemotherapeutic treatments. The future of kinase-targeted therapeutics in cancer appears promising, and implementation of these strategies will help to achieve therapeutic advances and overcome treatment hindrances.

## Conclusions

By transferring the γ-phosphate from the ATP-cofactor onto diverse substrates, kinases regulate key cellular functions. As many human diseases result from mutations and overexpression of kinases, this enzyme class symbolizes an important targeted strategy for drug development. Kinases also play indispensable roles in signaling pathways that regulate tumor cell functions. Deregulation of kinases leads to a variety of pathophysiological changes triggering cancer cell proliferation and metastases. Hyperactivation of kinases also increases anti-apoptotic effects. Currently, about one-third of all protein targets under research in the pharmaceutical industry are kinase-based. Kinase inhibitors represent targeted therapy resultant of the understanding of molecular genetics and molecular signaling pathways. Most of the FDA-approved kinase inhibitors target ATP binding site of kinase enzymes and display therapeutic indications against tumorigenesis. This class of therapeutics represents a transformation from conventional chemotherapy to targeted cancer treatment. Kinase inhibitors have overcome a major drawback of traditional cancer treatment as it effectively discriminates between normal non-malignant cells and rapidly proliferating cancer cells. This leads to fewer off-target effects and low toxicities in the cancer patient population. Kinase inhibitors are also often useful in combination with cytotoxic chemotherapy or radiation therapy. A vital challenge for clinical use of kinase inhibitors in the prevention of drug-resistant cancer stem cells. This phenomenon occurs due to cellular pressure to compensate for the loss of function of an important kinase. Pharmacogenomic factors including gene polymorphisms also contribute to primary kinase drug-resistance. Due to the clinical importance of kinase inhibitors, multiple strategies are required to overcome resistance mechanisms and develop more effective targeted therapies. A key approach is to allosterically induce and stabilize inactive kinase conformations. In the future, scientific advances may eventually allow scientists to combine mutagenesis screens through next generation sequencing and proteomic techniques with the computational modeling of compound interactions with all possible mutant variants of a targeted kinase. This will lead to the development of well-tolerated kinase inhibitors compared to traditional chemotherapeutic treatments. Overall, kinase inhibitors represent a new and promising approach to cancer therapy, one that is already providing beneficial clinical effects.

## Abbreviations

ABL: Abelson murine leukemia viral oncogene
Abl: Abelson murine leukemia viral oncogene homolog 1
Akt: Protein kinase B
ALK: Anaplastic lymphoma kinase
ATM: Ataxia telangiectasia mutated
Aur A & B: Aurora kinase A & B, B-Raf
BRAF: Proto-oncogene
BTK: Bruton agammaglobulinemia tyrosine kinase
CDK: Cyclin-dependent kinase
CHK1: Checkpoint kinase 1
c-Kit: Proto-oncogene c-Kit or Mast/stem cell growth factor receptor
c-Met: c-MET proto-oncogene
c-Ret: c-RET proto-oncogene
c-SRC: Proto-oncogene tyrosine-protein kinase
CTK: Cytoplasmic tyrosine kinase
c-YES: c-Yes proto-oncogene (pp62c-Yes)
EGFR: Epidermal growth factor receptor
ERBB2: V-Erb-B2 avian erythroblastic leukemia viral oncogene homolog
Fes: Feline sarcoma oncogene
FGFRs: Fibroblast growth factor receptors
Flt3, Flt-4: Fms-like tyrosine kinase 3, 4
HER-2: Human epidermal growth factor receptor-2
IGR-R: Insulin-like growth factor 1 receptor
IKK: IκB kinase
JAK2: Janus kinase 2
KIT: V-Kit hardy-zuckerman 4 feline sarcoma viral oncogene homolog
LK: Lipid kinase
MAPK: Mitogen-activated protein kinases
MEK: MEK kinase gene
mRCC: Metastatic renal cell carcinoma
mTOR: Mammalian target of rapamycin
NF-κB: Nuclear factor kappa-light-chain-enhancer of activated B cells
PDGFRs: Platelet-derived growth factor receptors
PDGFR-α: Platelet-derived growth factor receptor α
PDGFR-β: Platelet-derived growth factor receptor β
PI3K: Phosphatidylinositol-3-kinase
PI3KCA: Phosphatidylinositol-4,5-bisphosphate 3-kinase, catalytic subunit alpha
PIP3: Phosphatidylinositol-3, 4, 5-triphosphate,
PKCi: Protein kinase Ci
PLKs: Polo-like kinases
PTEN: Phosphatase and tensin homolog
RNAi: RNA interference
ROCK1: Rho-associated, coiled-coil-containing protein kinase 1
RON: Recepteur d’Origine Nantais
RSK2: Ribosomal protein kinase 2
RTK: Receptor tyrosine kinase
S/T Kinase: Serine/threonine kinase
S6K: Ribosomal protein S6 kinase
SGLT1: Sodium/glucose cotransporter 1
shRNA: A small hairpin RNA
SK1: Sphingosine kinase 1
SRC: Proto-oncogene tyrosine-protein kinase c
STK11/LKB1: Serine/threonine kinase 11 or liver kinase B1
Trkb: Tropomyosin-related kinase B
VEGFR-2: Vascular endothelial growth factor receptor 2
VEGFRs: Vascular endothelial growth factor receptors
